# Long-Term Weight Loss Outcomes Following Sleeve Gastrectomy and Their Association with Diet Quality, Postoperative Complications, and Sociodemographic Factors: A Retrospective Cohort Study in Jeddah, Saudi Arabia

**DOI:** 10.3390/jcm15124571

**Published:** 2026-06-12

**Authors:** Khalid A. Khormi, Walaa A. Mumena, Ahmed K. M. Salman, Ahmed A. Faden, Maryam S. Hafiz, Hebah A. Kutbi

**Affiliations:** 1 Clinical Nutrition Services, Erada and Mental Health Complex, Jeddah Second Health Cluster, Jeddah 23622, Saudi Arabia; khkhormi@moh.gov.sa; 2 Department of Clinical Nutrition, Faculty of Applied Medical Sciences, King Abdulaziz University, Jeddah 21589, Saudi Arabia; mshafiz@kau.edu.sa; 3 Clinical Nutrition Department, College of Applied Medical Sciences, Taibah University, Madinah 42353, Saudi Arabia; wmumena@taibahu.edu.sa; 4 Prince Sultan Center for Bariatric Surgery, King Fahd General Hospital, Jeddah 23325, Saudi Arabia; akm456@hotmail.com; 5 Department of General & Minimally Invasive Surgery, King Abdullah Medical Complex, Jeddah Second Health Cluster, Ministry of Health, Jeddah 23816, Saudi Arabia; ataher-faden@moh.gov.sa

**Keywords:** bariatric surgery, obesity, weight loss, diet quality, postoperative complications, Saudi Arabia

## Abstract

**Background/Objectives:** Bariatric surgery is an effective intervention for severe obesity; however, long-term outcomes may be influenced by postoperative dietary behaviors, nutritional status, and complications. In Saudi Arabia, longitudinal evidence on weight trajectories and postoperative diet quality remains limited. The present study aimed at evaluating three-year weight status trends; assessing sociodemographic factors, baseline BMI, and postoperative diet quality; and examining nutrition-related complications following bariatric surgery. **Methods:** This retrospective longitudinal study included 189 adults who underwent sleeve gastrectomy at two tertiary hospitals in Jeddah, Saudi Arabia. Anthropometric data were obtained from medical records at six time points: preoperative, two weeks, six months, one year, two years, and three years postoperatively. Diet quality and postoperative complications were assessed via structured telephone interviews. Weight outcomes were expressed as percentage of total body weight loss (%TBWL), excess body weight loss (%EWL), excess body mass index loss (%EBMIL), and weight regain. Statistical analyses included Friedman’s test, Mann–Whitney U test, and multiple linear regression. **Results:** Significant improvements in all weight loss indicators were observed over three years (*p* < 0.001). Diet quality score was the only significant variable associated with weight loss at three years, with higher scores associated with greater %EWL and %EBMIL. Baseline BMI and DQS were significantly associated with %EWL (Beta = −0.17, 95% CI: −1.72 to −0.13 and Beta = 0.21, 95% CI: 1.37 to 7.12, respectively) and %EBMIL (Beta = −0.15, 95% CI: −1.68 to −0.07 and Beta = 0.24, 95% CI: 1.90 to 7.66, respectively). Age was significantly associated with weight regain (Beta = 0.20, 95% CI: 0.02 to 1.08). **Conclusions:** Bariatric surgery resulted in sustained weight reduction over three years. Postoperative baseline BMI and diet quality were significantly associated with %EWL and %EBMIL, underscoring the importance of structured nutritional follow-up and counseling.

## 1. Introduction

Obesity is a complex, chronic disease characterized by the abnormal or excessive accumulation of body fat that adversely affects health. Excess adipose tissue also contributes to impaired reproductive function and other long-term health complications, highlighting obesity as a major global public health concern [1]. According to the World Health Organization, the prevalence of obesity has increased substantially over the past several decades due to major changes in dietary patterns, reduced physical activity, and rapid urbanization. In 2022, more than 2.5 billion adults aged 18 years and older were classified as overweight, including over 890 million adults living with obesity worldwide [2]. In Saudi Arabia, obesity prevalence has increased substantially over the past two decades [3]. A large nationwide cross-sectional study conducted by Althumiri et al. evaluated obesity prevalence among adults in Saudi Arabia and reported a national weighted prevalence of approximately 24.7% for obesity [4]. The study also demonstrated that obesity prevalence varied by region, age group, and sex. Some national analyses suggest that nearly two-thirds of adults in Saudi Arabia are either overweight or obese, reflecting a substantial public health burden for the healthcare system [5].

Severe obesity is associated with a wide range of adverse health outcomes affecting multiple physiological systems. Among the most significant consequences are cardiometabolic disorders, including type 2 diabetes mellitus, hypertension, dyslipidemia, and coronary artery disease [6,7]. Excess adiposity contributes to insulin resistance, endothelial dysfunction, and chronic low-grade inflammation, which together increase the risk of cardiovascular morbidity and mortality [8,9]. In addition to cardiometabolic complications, obesity is strongly associated with several other chronic conditions. These include non-alcoholic fatty liver disease, obstructive sleep apnea, osteoarthritis, and certain types of cancer such as colorectal, breast, and endometrial cancers [10,11,12,13,14].

Lifestyle modification remains the foundation of obesity management and typically includes dietary interventions, increased physical activity, and behavioral therapy. These strategies are widely recommended as first-line approaches for weight reduction and improving metabolic health [15,16]. However, although lifestyle interventions can produce modest short-term weight loss, maintaining these outcomes over the long term remains challenging for many individuals [17]. One major challenge in long-term weight management is physiological adaptation that occurs following weight loss [18]. These adaptations include reductions in resting metabolic rate and hormonal changes affecting appetite regulation, such as alterations in leptin and ghrelin levels. These physiological responses promote increased hunger and reduced energy expenditure, contributing to weight regain over time [19]. Consequently, bariatric surgery has emerged as the most effective intervention for achieving substantial and sustained weight loss in individuals with severe obesity [20]. Long-term studies have demonstrated that bariatric surgery results in significantly greater weight loss compared with medical or lifestyle management alone and is associated with improvements in obesity-related comorbidities and metabolic health outcomes [21].

Common procedures such as sleeve gastrectomy and Roux-en-Y gastric bypass are increasingly performed and have demonstrated favorable long-term outcomes in weight reduction and improvement of obesity-related conditions [22]. In Saudi Arabia, sleeve gastrectomy is the most performed procedure, followed by Roux-en-Y gastric bypass [23]. Weight loss following bariatric surgery is driven by a combination of restrictive, hormonal, metabolic, and behavioral mechanisms [24]. Gastric restriction reduces food intake, while hormonal changes such as decreased ghrelin and increased GLP-1 and PYY enhance satiety and regulate appetite [25]. Additionally, alterations in bile acid metabolism and gut microbiota composition contribute to improved metabolic regulation and energy balance [26,27]. These mechanisms, together with changes in dietary behavior, lead to substantial and sustained weight loss.

Evidence from systematic reviews and long-term cohort studies demonstrates sustained reductions in body weight, with many patients achieving clinically meaningful weight loss during the first one to three years postoperatively [28]. However, considerable heterogeneity in long-term outcomes has been reported across populations and procedures, with a substantial proportion of patients experiencing suboptimal weight loss or progressive weight regain over time [29]. Behavioral and clinical factors have increasingly been recognized as important determinants of long-term success following bariatric surgery [28]. In addition to the surgical procedure itself, factors such as dietary behaviors, adherence to postoperative nutritional recommendations, and the presence of postoperative complications may influence long-term weight trajectories and overall health outcomes [30,31]. Despite the high volume of bariatric procedures performed in Saudi Arabia, there remains a significant gap in context-specific evidence regarding long-term weight trajectories and their determinants. Limited local data are available examining the combined influence of diet quality, postoperative complications, and sociodemographic factors on sustained weight loss using multiple outcome indicators [30]. The absence of comprehensive longitudinal assessment within the Saudi bariatric population constrains the development of targeted postoperative management strategies aimed at optimizing long-term success and mitigating weight regain. Therefore, this study aims to address this gap by evaluating weight status over a three-year postoperative period, assessing diet quality using a modified short-form food frequency questionnaire, and documenting the occurrence of post-operative complications at multiple follow-up time points.

## 2. Materials and Methods

### 2.1. Study Design

This study employed a retrospective cohort design to evaluate long-term weight loss outcomes following bariatric surgery and to examine the influence of diet quality, postoperative complications, and sociodemographic factors on these outcomes. The study was conducted in Jeddah, Saudi Arabia, at King Abdullah Medical Complex and King Fahad General Hospital. Eligible patients were identified through hospital medical records, and data were collected for individuals who had completed a three-year postoperative follow-up period.

Data collection included retrospective extraction of clinical and anthropometric variables from medical records at predefined postoperative time points, in addition to telephone interviews to assess postoperative complications and diet quality. The study was conducted in accordance with the Declaration of Helsinki, and the protocol was approved by the Institutional Review Board of Jeddah, Ministry of Health (Certificate No. A02049) on 20 November 2024. Patient confidentiality was strictly maintained, and all data were de-identified prior to analysis and stored on secure, password-protected systems.

### 2.2. Participants and Recruitment 

Eligible participants were identified through hospital electronic medical record systems at the participating hospitals. Medical records of all patients who underwent bariatric surgery during the study period and had completed at least three years of postoperative follow-up were screened for eligibility. The study included adult patients aged 18 years or older who underwent laparoscopic sleeve gastrectomy and had available anthropometric records across the predefined postoperative follow-up period.

Patients were excluded if they had incomplete baseline anthropometric data, missing key follow-up weight records, had undergone revisional bariatric procedures, or had major medical conditions affecting metabolism such as malignancy or severe renal disease. Patients who were pregnant or lactating during the study period were also excluded.

Patients who met the eligibility criteria were contacted by telephone and invited to participate in the interview component of the study. Verbal informed consent was obtained prior to conducting the telephone interview.

### 2.3. Sample Size Calculation

Sample size estimation was informed by previous studies reporting substantial weight loss following bariatric surgery. The calculation was based on the primary study outcome of postoperative weight-related changes and the planned regression analyses examining associations with all independent variables (marital status, education level, baseline BMI, and diet quality). The minimum sample size needed for this study, calculated using G*Power version 3.1.9.7 with f^2^ = 0.15, 80% power, and alpha of 0.05, was 98 adults who underwent bariatric surgery during the study period and had completed at least three years of postoperative follow-up.

### 2.4. Data Collection Methods and Instruments

Data were collected from two primary sources: electronic medical records and structured telephone questionnaires. Anthropometric data, including weight in kg and height in cm, were extracted from electronic medical records. Body mass index (BMI) was calculated using the standard formula (kg/m^2^).

Weight status was assessed at six time points: preoperative, two weeks, six months, one year, two years, and three years post-surgery. In addition to absolute weight and BMI, %TBWL, %EWL, %EBMIL and weight regain were calculated to quantify the magnitude of weight change over time.

%TBWL was calculated as the difference between preoperative weight and postoperative weight divided by preoperative weight, multiplied by 100. %EWL was calculated using ideal body weight corresponding to a BMI of 25 kg/m^2^ as the reference standard. %EBMIL was calculated by dividing the amount of BMI reduction after surgery by the amount of excess BMI before surgery. Weight regain was calculated as an increment in body weight following descending to the lowest postoperative weight (nadir), expressed as a percentage of maximum weight lost.

Diet quality was assessed using the modified short-form food frequency questionnaire, originally developed by Cleghorn et al. [32,33]. The questionnaire has previously been validated and used in different populations [33]. The modified version underwent content validity by two experts in dietary assessment to verify the inclusion of commonly consumed foods and to ensure the questionnaire’s relevance to the Saudi population. In addition, the questionnaire was reviewed for clarity and suitability for telephone administration. The questionnaire was administered via telephone interview.

Participants were asked to report their habitual dietary intake over the past month. The questionnaire included items related to the consumption of fruits, fruit juice, salad, cooked vegetables, fried potatoes/chips, beans or legumes, fiber-rich breakfast cereals, whole wheat bread, cheese/yoghurt, crisps or savory snacks, sweet biscuits, ice cream/cream, and fizzy drinks/soda. Response options for these items ranged from “rarely or never” to “≥5 times/day.” Additional items assessed the intake of beef or lamb, chicken or turkey, processed meat products, processed chicken or turkey products, battered white fish products, white fish, and oily fish. Response options for these items ranged from “rarely or never” to “≥7 times/week”. Consumption frequencies were entered into the Nutritools spreadsheet tool to automatically calculate the diet quality score (DQS) based on five dietary components: fruit intake, vegetable intake, oily fish consumption, total dietary fat, and non-milk extrinsic sugars. Each component was scored using a three-point scoring system ranging from 1 to 3, reflecting adherence to dietary recommendations. The component scores were then summed to generate a DQS ranging from 5 to 15, with higher scores indicating better overall diet quality [32]. The adapted questionnaire was reviewed for clarity and suitability for telephone administration prior to data collection.

Post-operative complications were self-reported using a structured questionnaire adapted from the work published by Hassan et al. The questionnaire was used to identify the occurrence and prevalence of common post-operative complications, including dehydration, constipation, hair loss, and related symptoms [34]. Complications were assessed at five postoperative time points: two weeks, six months, one year, two years, and three years after surgery.

### 2.5. Statistical Analysis

Data were entered and coded using Microsoft Excel and analyzed using IBM SPSS Statistics (version 20.0). Normality of continuous variables was assessed using the Shapiro–Wilk test, which indicated that the majority of continuous variables were not normally distributed (*p* < 0.05). Accordingly, continuous variables are presented as median with interquartile range (IQR) in addition to mean ± standard deviation (SD), while categorical variables are summarized as frequencies and percentages. Friedman’s test for repeated measures was used to examine longitudinal changes in %EWL, %TBWL, and %EBMIL at 6-point. Wilcoxon Signed-Rank test was used to compare %EWL, %TBWL, and %EBMIL between time points. Bonferroni correction was used to adjust for multiple testing (10 comparisons for each outcome).

Multiple linear regression analysis was performed to identify factors associated with weight change indicators at three years postoperatively (independent variables: age (in years), sex, marital status, education level, baseline BMI in kg/m^2^, and DQS). Mann–Whitney U test was used to explore the associations of complications with weight change and diet quality. A two-sided significance level of α = 0.05 was adopted for all analyses, and exact *p*-values were reported.

## 3. Results

A total of 250 patient records were screened for eligibility. Of these, 61 patients were excluded due to declined participation, failure to respond to telephone contact attempts, undergoing additional surgical procedures during the follow-up period, use of medications known to affect weight loss, pregnancy during the previous three years, or death. The final study sample consisted of 189 participants who met the eligibility criteria and completed the study assessment. After exclusion, a total of 189 patients who underwent sleeve gastrectomy were included in this study. The mean age of the participants was 36.6 ± 10.7 years, with a median age of 36.0 years (IQR: 28.5–44.0). Females represented the majority of the sample (66.7%).

Regarding educational status, approximately 70% of the participants held a university degree, while 57.7% were married. The mean preoperative body weight was 113 ± 17.1 kg, with a median of 113 kg (IQR: 102–121). The mean baseline BMI was 42.7 ± 5.18 kg/m^2^, with a median of 42.0 kg/m^2^ (IQR: 39.2–46.0).

Most participants (*n* = 133, 70.4%) were classified as having class III obesity at baseline, indicating severe obesity. Detailed sociodemographic and baseline clinical characteristics of the study sample are presented in Table 1.

### 3.1. Longitudinal Changes in Weight Loss Outcomes

Longitudinal changes in %EWL, %TBWL, and %EBMIL across the postoperative follow-up periods are presented in Table 2. A significant increase in the median values of %EWL, %TBWL, and %EBMIL was observed across the five postoperative time points (2 weeks, 6 months, 1 year, 2 years, and 3 years), *p* < 0.001 for all comparisons.

Pairwise Wilcoxon Signed-Rank post hoc comparisons with Bonferroni correction showed significant increases in all weight-loss parameters from 2 weeks to each subsequent follow-up time point, and from 6 months to 1, 2, and 3 years (%EWL, %TBWL, and %EBMIL; adjusted *p* ≤ 0.010). For %EWL, significant increases were also observed from 1 year to 2 years and from 1 year to 3 years, while the difference between 2 and 3 years was not significant. For %TBWL, differences after 1 year were no longer statistically significant after adjustment, including 1 year vs. 2 years, 1 year vs. 3 years, and 2 years vs. 3 years. For %EBMIL, the increase from 1 year to 2 years remained significant, whereas 1 year vs. 3 years and 2 years vs. 3 years were not significant after Bonferroni correction (Table S1).

### 3.2. Factors Associated with Weight Change Outcomes at Three Years Postoperatively

Multiple linear regression analysis was performed to identify factors associated with weight change outcomes at three years postoperatively (Table 3). Baseline BMI and DQS were significantly associated with %EWL (Beta = −0.17, 95% CI: −1.72 to −0.13 and Beta = 0.21, 95% CI: 1.37 to 7.12, respectively) and %EBMIL (Beta = −0.15, 95% CI: −1.68 to −0.07 and Beta = 0.24, 95% CI: 1.90 to 7.66, respectively). Age was significantly associated with weight regain (Beta = 0.20, 95% CI: 0.02 to 1.08). No associated factors were identified for %TBWL at three years postoperatively.

### 3.3. Postoperative Complications

The Incidence of postoperative complications reported by participants during the three-year postoperative period is presented in Table 4. A range of gastrointestinal, nutritional, and general symptoms were reported by the study participants. The most frequently reported complications included hair loss, fatigue, gastroesophageal reflux disease (GERD), and constipation. Other reported complications included diarrhea, abdominal pain or flatulence, dry skin, anemia, lactose intolerance, dysphagia, hypoglycemia, and reduced appetite.

The association between postoperative complications and weight loss outcomes at three years after surgery was examined using the Mann–Whitney U test. Weight loss indicators, including %EWL, %TBWL, and %EBMIL, were compared between patients who reported each complication and those who did not. Overall, no statistically significant differences in weight loss outcomes were observed according to the presence or absence of the reported postoperative complications (Table S2).

### 3.4. Association Between Diet Quality and Postoperative Complications at Three Years

The association between DQS and postoperative complications at three years after surgery was examined using the Mann–Whitney U test (Table 4). No statistically significant differences in DQS were observed for the reported complications.

## 4. Discussion

This study examined longitudinal weight changes, diet quality, and postoperative complications among patients who underwent sleeve gastrectomy over a three-year follow-up period. Overall, the findings demonstrate that bariatric surgery resulted in substantial and statistically significant reductions in body weight, BMI, and excess weight indicators across the postoperative period. The greatest reduction in weight occurred during the early postoperative period, particularly between two weeks and one year after surgery, after which the rate of change slowed, and weight loss stabilized. This trajectory is consistent with findings reported in previous bariatric surgery studies. Long-term research indicates that the majority of weight reduction occurs within the first one to two years following surgery [35]. Budny et al. (2024) reported that many patients achieve excess weight loss exceeding 50% within five years after bariatric surgery [36]. Similarly, Karpińska et al. (2021) observed that patients typically experience rapid weight reduction during the first postoperative year [37].

Regional studies also support these findings. Alamri et al. (2024) reported significant reductions in body weight within three to six months after surgery among Saudi patients [38]. Likewise, Alfadda et al. (2021) demonstrated that weight loss indicators increased progressively and peaked around the third postoperative year in a Saudi bariatric cohort [39]. The observed trajectory may be explained by physiological and behavioral mechanisms following sleeve gastrectomy. Surgical restriction reduces stomach capacity and limits food intake, while hormonal changes involving GLP-1 and peptide YY contribute to increased satiety and reduced appetite [40]. During the early postoperative period, patients also tend to adhere strictly to dietary guidelines, which further promotes rapid weight loss [41]. Over time, however, metabolic adaptation and gradual changes in dietary behaviors may slow the rate of weight reduction [42]. Patients may progressively increase caloric intake or deviate from recommended dietary patterns, leading to stabilization of weight loss or partial weight regain. This pattern of rapid early weight loss followed by stabilization has been widely documented in bariatric literature and reflects the combined influence of surgical effects and long-term behavioral adaptation [43]. Nevertheless, these findings confirm that sleeve gastrectomy is highly effective in producing substantial weight reduction over a three-year period, although the rate of weight loss tends to decline as postoperative follow-up progresses.

Diet quality following bariatric surgery represents an important factor correlating with long-term health outcomes [39]. In the present study, diet quality was assessed using a modified short-form food frequency questionnaire, which enabled the calculation of a DQS reflecting overall dietary patterns and nutritional adequacy. Analysis of the current study showed that patients with higher diet quality scores demonstrated greater %EWL and %EBMIL at three years postoperatively. These findings suggest that healthier dietary patterns correlate with more favorable long-term weight outcomes. Previous research has similarly emphasized the role of dietary behaviors in postoperative success. Zarshenas et al. (2020) reported that many bariatric patients develop suboptimal dietary patterns in the long term, characterized by inadequate intake of protein and nutrient-dense foods and increased consumption of energy-dense foods [44]. Such dietary patterns may contribute to nutritional deficiencies and reduce the effectiveness of bariatric surgery. Hosseini-Esfahani et al. (2023) also found that despite significant weight loss following surgery, many patients demonstrate poor adherence to recommended dietary patterns, including high intake of saturated fats and sodium and insufficient consumption of fruits and vegetables [45].

The association observed between higher DQS and improved excess weight indicators in the present study may reflect better adherence to postoperative nutritional recommendations. Previous studies have shown that adherence to recommended dietary patterns after bariatric surgery is associated with more favorable weight outcomes and improved long-term weight management [46]. This suggests that overall dietary quality, rather than specific dietary components, may play an important role in supporting sustained weight reduction following bariatric surgery. These findings highlight the importance of continuous nutritional monitoring and counseling after bariatric surgery. Although surgery provides a powerful physiological tool for weight reduction, long-term success appears to depend partly on patients’ ability to maintain healthy dietary habits.

Postoperative complications were commonly reported among participants during the three-year follow-up period. The most frequently reported symptoms included hair loss, fatigue, gastroesophageal reflux disease, and constipation. These findings are consistent with previous literature indicating that gastrointestinal and nutritional symptoms are common following bariatric surgery. For instance, one study reported high prevalence of hair loss, reflux symptoms, and vomiting among post-bariatric surgery patients [47]. Similarly, Hasan et al. (2020) observed similar complications including hair loss, abdominal discomfort, and dry skin in patients undergoing bariatric procedures in the Middle East [34]. Many of these complications are related to physiological changes following surgery. For example, hair loss has been associated with rapid weight loss and micronutrient deficiencies, particularly deficiencies in iron, zinc, and protein. Zhang et al. (2021) reported that hair loss occurs in approximately 57% of bariatric patients, particularly during the first year after surgery [48]. Long-term studies have shown that deficiencies in iron, folate, and vitamin B12 may increase following bariatric surgery, particularly in patients who do not adhere to supplementation recommendations [49].

Despite the relatively high prevalence of postoperative complications in the present study, no significant association was observed between these symptoms and long-term weight loss indicators. This suggests that while postoperative symptoms may affect patient comfort and quality of life, they may not necessarily influence the overall magnitude of weight loss.

This research has several strengths. First, the use of a retrospective longitudinal cohort design allowed for the evaluation of weight trajectories over multiple standardized postoperative time points, providing a comprehensive assessment of long-term outcomes after surgery. Second, to the best of our knowledge, this is among the first studies conducted in Saudi Arabia, particularly in the Jeddah region, to simultaneously examine weight trajectories, diet quality, and postoperative complications within the same cohort. Third, the use of multiple weight change parameters including %TBWL, %EWL, %EBMIL, and %weight regain provided a more robust and multidimensional evaluation of postoperative weight outcomes compared to reliance on a single metric.

On the other hand, some limitations should be acknowledged. Dietary intake and postoperative complications were assessed retrospectively using telephone interviews, which introduces the possibility of recall bias, affects the accuracy of self-reported data and limits the ability to establish temporal directionality or causal relationships; hence, the possibility of reverse causation cannot be excluded. Furthermore, the study was conducted in two public hospitals within a single city and included only patients with available anthropometric records and at least three years of postoperative follow-up, which may limit the generalizability of the findings to other regions, healthcare settings, or populations with different demographic and clinical characteristics in terms of adherence, lifestyle behaviors, or postoperative outcomes. In addition, the study used a modified short-form food frequency questionnaire to assess diet quality. Although the questionnaire was adapted for cultural relevance and reviewed for content validity, formal validation was not conducted specifically among post-bariatric Saudi patients. Therefore, the DQS may not fully capture dietary behaviors unique to bariatric populations. Specifically, DQS primarily reflects general dietary patterns and does not assess several post-bariatric nutritional factors, including protein intake adequacy, adherence to vitamin and mineral supplementation, hydration practices, meal frequency, or dumping-related eating behaviors. Future studies should consider the development and validation of bariatric-specific dietary assessment tools tailored to the Saudi population. Moreover, the study lacks assessment of behavioral and psychological factors that may influence long-term postoperative outcomes, such as emotional eating, binge eating behaviors, psychological support, and adherence to postoperative follow-up. The absence of these variables may have resulted in residual confounding and limits the comprehensive interpretation of factors associated with postoperative weight outcomes. Another limitation of this study is the absence of biochemical assessment of postoperative nutritional status. Although participants reported symptoms such as fatigue, hair loss, dry skin, and anemia, no laboratory measurements of key nutritional markers (e.g., iron, ferritin, vitamin B12, folate, zinc, vitamin D, or protein status) were available; therefore, interpretations regarding nutritional deficiencies remain speculative.

Findings of the present study suggest several implications for clinical practice and healthcare services involved in the management of patients undergoing bariatric surgery. Strengthening long-term postoperative follow-up programs is essential to ensure sustained weight management after surgery. Regular monitoring of weight, BMI, and other weight-related indicators across multiple postoperative time points can help clinicians identify patterns of weight change, detect early signs of weight regain, and provide timely interventions when necessary. Establishing structured follow-up schedules within bariatric clinics may therefore support improved long-term outcomes. In particular, clinical nutrition services should play a central role in postoperative care by providing continuous dietary counseling and monitoring. Given the observed association between better diet quality and favorable weight loss outcomes, dietitians should guide patients in maintaining balanced dietary patterns that support adequate nutrient intake while controlling total energy consumption. Nutritional education should emphasize appropriate portion sizes, adequate protein intake, and the inclusion of nutrient-dense foods to support both weight management and nutritional adequacy after surgery.

Healthcare providers should also maintain regular assessment of postoperative symptoms and nutritional concerns that may arise during long-term follow-up. Symptoms such as fatigue, hair loss, gastrointestinal discomfort, or constipation may influence patient wellbeing and adherence to dietary recommendations. Routine evaluation of these symptoms, together with appropriate nutritional assessment, can help identify potential deficiencies or dietary imbalances and allow early clinical management when necessary. Multicenter cohort studies conducted across different regions of Saudi Arabia would provide more representative evidence regarding postoperative weight changes and help clarify variations in weight loss patterns across populations, surgical techniques, and healthcare settings. Future research should also further explore the relationship between dietary patterns and long-term weight outcomes after bariatric surgery, incorporating comprehensive behavioral, psychological, and lifestyle assessments, including eating behaviors, physical activity, mental health status, and adherence to postoperative follow-up, to better understand their influence on long-term bariatric surgery outcomes. Longitudinal studies integrating both clinical and psychosocial variables are warranted to provide a more comprehensive evaluation of factors associated with postoperative success. While this study demonstrated an association between higher DQS and improved excess weight loss indicators, additional prospective studies are needed to examine how specific dietary components, eating behaviors, and adherence to nutritional recommendations influence weight maintenance and metabolic outcomes after surgery.

## 5. Conclusions

Bariatric surgery was associated with significant and sustained weight loss over a three-year follow-up period. Baseline BMI and postoperative diet quality emerged as significant correlates of long-term weight loss outcomes, emphasizing the important role of healthy dietary behaviors in maintaining surgical benefits. Although postoperative complications were frequently reported, they were not significantly associated with weight loss. These findings underscore the need for structured nutritional follow-up, individualized dietary counseling, and long-term postoperative monitoring to optimize outcomes after bariatric surgery.

## Figures and Tables

**Table 1:** Sociodemographic and Baseline Clinical Characteristics of the Study Participants (*n* = 189).

Characteristic	Category	*n* (%)
Gender	Male	63 (33.3)
	Female	126 (66.7)
Age	18–28 years	47 (24.9)
	29–39 years	68 (36.0)
	40–50 years	56 (29.6)
	≥50 years	18 (9.50)
Marital status	Single	80 (42.3)
	Married	109 (57.7)
Education level	Secondary school or less	56 (29.6)
	University degree	133 (70.4)
Baseline weight status	Obesity class I (30.0–34.9 kg/m^2^)	11 (5.80)
	Obesity class II (35.9–39.9 kg/m^2^)	45 (23.8)
	Obesity class III (≥40 kg/m^2^)	133 (70.4)

**Table 2:** Longitudinal Changes in Weight Loss Indicators Across Postoperative Time Points.

Time Point	%EWL Median (IQR)	%TBWL Median (IQR)	%EBMIL Median (IQR)
2 weeks	18.8 (13.5–27.1)	7.41 (5.59–9.64)	19.1 (11.9–27.2)
6 months	51.5 (39.4–71.2)	20.6 (15.0–26.8)	49.9 (38.0–63.5)
1 year	71.4 (56.0–87.9)	30.1 (22.8–37.0)	72.9 (61.0–87.9)
2 years	81.6 (66.7–96.2)	35.4 (27.0–40.6)	79.1 (67.9–93.4)
3 years	86.5 (67.1–98.4)	33.6 (25.3–40.7)	82.2 (66.5–98.3)
***p*-value**	**<0.001** *	**<0.001** *	**<0.001** *

* Statistically significant at 95% confidence level. Friedman’s test for repeated measures was used. Abbreviations: EWL, excess body weight loss; TBWL, total body weight loss; EBMIL, excess body mass index loss; IQR, interquartile range.

**Table 3:** Multiple Linear Regression Analysis of Factors Associated with Weight Change Indicators at Three Years Postoperatively.

Outcome Variable	Factors	R^2^	B (SE)	Beta	95% Confidence Interval	*p*-Value
%EWL		0.08				
	Age, years		−0.29 (0.26)	−0.11	−0.79 to 0.22	0.264
	Sex		0.82 (4.50)	0.01	−8.05 to 9.69	0.855
	Marital status		3.19 (5.46)	0.05	−13.2 to 5.91	0.560
	Education level		−3.64 (4.84)	−0.06	−13.2 to 5.91	0.453
	Baseline BMI, kg/m^2^		−0.92 (0.40)	−0.17	−1.72 to −0.13	0.023 *
	Diet quality score		4.24 (1.46)	0.21	1.37 to 7.12	0.004 *
%TBWL		0.02				
	Age, years		0.16 (0.17)	0.09	−0.19 to 0.50	0.364
	Sex		−3.11 (3.10)	−0.08	−9.23 to 3.01	0.318
	Marital status		−0.99 (3.81)	−0.03	−8.52 to 6.54	0.796
	Education level		3.86 (3.42)	0.09	−2.89 to 10.6	0.261
	Baseline BMI, kg/m^2^		−0.12 (0.28)	−0.03	−0.67 to 0.44	0.679
	Diet quality score		−0.75 (1.07)	−0.06	−2.86 to 1.37	0.487
%EBMIL		0.09				
	Age, years		−0.39 (0.26)	−0.14	−0.90 to 0.11	0.128
	Sex					0.888
	Marital status		3.51 (5.47)	0.06	−7.29 to 14.3	0.522
	Education level		−5.96 (4.89)	−0.09	−15.6 to 3.68	0.224
	Baseline BMI, kg/m^2^		−0.88 (0.41)	−0.15	−1.68 to −0.07	0.034 *
	Diet quality score		4.78 (1.46)	0.24	1.90 to 7.66	0.001 *
%Weight regain		0.03				
	Age, years		0.55 (0.27)	0.20	0.02 to 1.08	0.041 *
	Sex		−0.22 (4.69)	−0.01	−9.47 to 9.04	0.963
	Marital status		−9.97 (5.70)	−0.17	−21.2 to 1.26	0.082
	Education level		−0.45 (5.05)	−0.01	−10.4 to 9.52	0.930
	Baseline BMI, kg/m^2^		−0.37 (0.42)	−0.06	−1.20 to 0.47	0.387
	Diet quality score		−0.79 (1.52)	−0.04	−3.79 to 2.21	0.602

Abbreviations: EWL, excess body weight loss; TBWL, total body weight loss; EBMIL, excess body mass index loss. All models met all assumptions of multiple regression analysis (there is no multicollinearity in the data: tolerance value above 0.2 and VIF below 10; the values of the residuals are independent: Durbin–Watson value close to 2; the variance of the residuals is constant; the values of the residuals are normally distributed; there are no influential cases biasing the models). * Statistically significant at *p* < 0.050.

**Table 4:** Associations Between Diet Quality Score and Postoperative Complications at 3 Years Postoperatively.

Complication	Incidence	No Median (IQR)	Yes Median (IQR)	*p*-Value
Dry skin	66.1%	11.0 (10.0–12.0)	10.0 (9.0–12.0)	0.032
Constipation	73.0%	10.0 (10.0–12.0)	10.0 (9.0–12.0)	0.842
Diarrhea	23.4%	11.0 (9.0–12.0)	10.0 (9.25–11.0)	0.211
Dysphagia	15.3%	10.0 (10.0–12.0)	10.0 (9.0–11.0)	0.400
Low appetite	56.6%	10.0 (9.0–12.0)	10.0 (10.0–12.0)	0.788
Hair loss	83.1%	10.5 (10.0–12.0)	10.0 (9.0–12.0)	0.575
Abdominal pain/flatulence	43.1%	11.0 (10.0–12.0)	10.0 (9.0–12.0)	0.296
Anemia	53.4%	11.0 (10.0–12.0)	10.0 (9.0–12.0)	0.059
Hypotension	31.2%	10.5 (10.0–12.0)	10.0 (9.0–12.0)	0.431
Hypoglycemia	24.3%	11.0 (9.0–12.0)	10.0 (10.0–11.3)	0.451
Lactose intolerance	16.4%	10.0 (9.0–12.0)	11.0 (10.0–12.0)	0.787
GERD	69.3%	10.0 (9.0–12.0)	10.0 (9.0–12.0)	0.864
Cholelithiasis	9.00%	10.0 (9.0–12.0)	10.0 (9.5–11.0)	0.162
Fatigue	63.0%	11.0 (10.0–12.0)	10.0 (9.0–11.0)	0.013

Mann–Whitney U test was used to examine differences across groups. Significance denoted at *p* < 0.004 based on Bonferroni adjustment. Abbreviations: IQR, interquartile range; GERD, gastroesophageal reflux disease.

## Data Availability

Data presented in this study are available upon request from the corresponding author due to privacy and ethical purposes.

## Supplementary Materials

The following supporting information can be downloaded at https://www.mdpi.com/article/10.3390/jcm15124571/s1. Table S1: Pairwise comparisons of weight loss parameters between time points; Table S2: Incidence of postoperative complications and the association with weight change at 3 years.

## Abbreviations

The following abbreviations are used in this manuscript:

%TBWL	Percentage total body weight loss
%EWL	Percentage of excess body weight loss
%EBMIL	Percentage of excess body mass index loss
BMI	Body mass index
DQS	Diet quality score
SD	Standard deviation
IQR	Interquartile range
GERD	Gastroesophageal reflux disease
