# Supplementary material for: Long-Term Weight Loss Outcomes Following Sleeve Gastrectomy and Their Association with Diet Quality, Postoperative Complications, and Sociodemographic Factors: A Retrospective Cohort Study in Jeddah, Saudi Arabia
Source: J Clin Med. 2026 Jun 12;15(12):4571. doi: 10.3390/jcm15124571 (PMC13301076; doi:10.3390/jcm15124571)
Supplement: Supplementary file 1 [file jcm-15-04571-s001.zip › jcm-4290252-supplementary.pdf]

Table S1. Pairwise comparisons of weight loss parameters between time points.

Variable	Time point 1 Median (IQR)	Time point 2 Median (IQR)	*Z*	*Unadjusted p-value*	*Adjusted p-value*
**%EWL**					
2 weeks vs. 6 months	18.8 (13.5-27.1)	51.5 (39.4-71.2)	-11.3	< 0.001	0.010*
2 weeks vs. 1 year		71.4 (56.0-87.9)	-11.6	< 0.001	0.010*
2 weeks vs. 2 years		81.6 (66.7-96.2)	-11.7	< 0.001	0.010*
2 weeks vs. 3 years		86.5 (67.1-98.4)	-11.6	< 0.001	0.010*
6 months vs. 1 year	51.5 (39.4-71.2)	71.4 (56.0-87.9)	-7.03	< 0.001	0.010*
6 months vs. 2 years		81.6 (66.7-96.2)	-9.11	< 0.001	0.010*
6 months vs. 3 years		86.5 (67.1-98.4)	-8.89	< 0.001	0.010*
1 year vs. 2 years	71.4 (56.0-87.9)	81.6 (66.7-96.2)	-4.53	< 0.001	0.010*
1 year vs. 3 years		86.5 (67.1-98.4)	-4.12	< 0.001	0.010*
2 years vs. 3 years	81.6 (66.7-96.2)	86.5 (67.1-98.4)	-1.00	0.317	1.00
**%TBWL**					
2 weeks vs. 6 months	7.41 (5.59-9.64)	20.6 (15.0-26.8)	-7.85	< 0.001	0.010*
2 weeks vs. 1 year		30.1 (22.8-37.0)	-7.66	< 0.001	0.010*
2 weeks vs. 2 years		35.4 (27.0-40.6)	-7.62	< 0.001	0.010*
2 weeks vs. 3 years		33.6 (25.3-40.7)	-8.78	< 0.001	0.010*
6 months vs. 1 year	20.6 (15.0-26.8)	30.1 (22.8-37.0)	-4.26	< 0.001	0.010*
6 months vs. 2 years		35.4 (27.0-40.6)	-5.24	< 0.001	0.010*
6 months vs. 3 years		33.6 (25.3-40.7)	-6.62	< 0.001	0.010*
1 year vs. 2 years	30.1 (22.8-37.0)	35.4 (27.0-40.6)	-2.64	0.008	0.080
1 year vs. 3 years		33.6 (25.3-40.7)	-1.58	0.114	1.00
2 years vs. 3 years	35.4 (27.0-40.6)	33.6 (25.3-40.7)	-0.03	0.978	1.00
**%EBMIL**					
2 weeks vs. 6 months	19.1 (11.9-27.2)	49.9 (38.0-63.5)	-11.2	< 0.001	0.010*
2 weeks vs. 1 year		72.9 (61.0-87.9)	-11.8	< 0.001	0.010*
2 weeks vs. 2 years		79.1 (67.9-93.4)	-11.7	< 0.001	0.010*
2 weeks vs. 3 years		82.2 (66.5-98.3)	-11.7	< 0.001	0.010*
6 months vs. 1 year	49.9 (38.0-63.5)	72.9 (61.0-87.9)	-9.19	< 0.001	0.010*
6 months vs. 2 years		79.1 (67.9-93.4)	-9.53	< 0.001	0.010*
6 months vs. 3 years		82.2 (66.5-98.3)	-9.09	< 0.001	0.010*
1 year vs. 2 years	72.9 (61.0-87.9)	79.1 (67.9-93.4)	-3.48	0.001	0.010*
1 year vs. 3 years		82.2 (66.5-98.3)	-2.12	0.034	0.340
2 years vs. 3 years	79.1 (67.9-93.4)	82.2 (66.5-98.3)	-0.15	0.878	1.00

Pairwise Wilcoxon signed-rank test was used. * Significant at the 95% confidence level. Bonferroni correction was conducted to adjust for multiple testing.

Abbreviations: EWL, excess body weight loss; TBWL, total body weight loss; EBMIL, excess body mass index loss

Table S2. Incidence of postoperative complications and the association with weight change at 3 years.

Complication	Incidence	EWL%		TBWL%		EBMIL%	
		Median (IQR)	*p- value*	Median (IQR)	*p- value*	Median (IQR)	*p- value*
**Dry skin**							
No	66.1%	79.9 (63.3-97.7)	0.088	34.1 (28.7-41.4)	0.066	76.9 (59.0-96.5)	0.140
Yes		89.5 (67.8-99.0)		32.9 (21.6-39.8)		86.9 (67.3-98.6)	
**Constipation**							
No	73.0%	94.1 (67.3-102)	0.451	32.0 (24.6-37.0)	0.176	93.2 (67.3-100)	0.370
Yes		84.4 (66.8-98.3)		34.1 (25.5-41.2)		80.9 (65.4-97.0)	
**Diarrhea**							
No	23.4%	86.8 (70.1-98.8)	0.420	34.0 (27.1-41.0)	0.300	84.3 (67.3-98.5)	0.679
Yes		83.4 (59.6-97.7)		31.8 (21.9-39.3)		80.7 (60.2-97.3)	
**Dysphagia**							
No	15.3%	87.8 (68.7-99.1)	0.238	33.7 (24.1-40.7)	0.990	85.3 (67.3-98.5)	0.170
Yes		80.5 (59.7-95.7)		32.7 (26.9-39.3)		79.7 (50.7-94.9)	
**Low appetite**							
No	56.6%	86.3 (68.3-98.2)	0.828	33.0 (22.7-39.8)	0.158	85.0 (66.9-98.3)	0.866
Yes		86.5 (64.6-99.7)		33.7 (27.2-43.3)		80.5 (64.5-98.0)	
**Hair loss**							
No	83.1%	89.0 (53.9-101)	0.986	34.0 (28.6-42.4)	0.504	86.8 (51.4-99.5)	0.981
Yes		85.3 (67.8-98.2)		33.0 (24.1-40.6)		81.7 (67.1-98.3)	
**Abdominal pain flatulence**							
No	43.1%	89.2 (71.1-98.4)	0.429	33.9 (27.8-40.5)	0.535	86.7 (65.7-98.3)	0.355
Yes		81.8 (66.4-98.6)		33.0 (20.8-42.8)		79.7 (66.6-95.0)	
**Anemia**							
No	53.4%	89.8 (70.5-101)	0.261	33.6 (24.1-40.7)	0.914	89.0 (70.4-100)	0.071
Yes		84.1 (65.3-97.7)		33.6 (26.5-41.7)		78.7 (58.5-96.7)	
**Hypotension**							
No	31.2%	89.2 (66.5-100)	0.472	33.6 (25.6-41.0)	0.747	83.2 (62.7-98.5)	0.865
Yes		85.3 (68.3-96.8)		33.7 (22.6-40.7)		80.9 (68.4-97.0)	
**Hypoglycemia**							
No	24.3%	85.3 (68.3-98.1)	0.851	33.7 (25.6-41.0)	0.328	81.1 (66.2-98.0)	0.567
Yes		86.5 (62.3-102)		31.9 (24.3-39.7)		86.4 (66.2-102)	
**Lactose intolerance**							
No	16.4%	86.8 (66.6-98.6)	0.990	33.6 (25.6-40.4)	0.962	84.7 (66.6-98.6)	0.403
Yes		84.2 (70.0-98.3)		30.1 (18.7-43.4)		78.3 (63.9-94.7)	
**Gastro esophageal reflux disease**							
No	69.3%	90.5 (75.3-100)	0.098	33.0 (18.8-41.0)	0.523	89.5 (72.1-99.7)	0.153
Yes		83.0 (64.6-97.9)		33.6 (28.0-40.7)		80.6 (62.6-97.5)	
**Cholelithiasis**							
No	9.00%	86.5 (67.9-98.3)	0.664	33.3 (24.2-40.7)	0.254	84.3 (66.8-98.3)	0.242
Yes		71.8 (57.6-108)		37.1 (30.4-45.5)		71.2 (38.3-97.0)	
**Fatigue**							
No	63.0%	89.0 (67.0-102)	0.706	33.0 (25.6-40.4)	0.622	89.6 (65.3-101)	0.305
Yes		85.3 (66.9-98.0)		34.0 (24.7-43.0)		80.6 (66.4-97.0)	

Mann-Whitney U test was used to examine differences across groups. * Significant at the 95% confidence level.

Abbreviations: EWL, excess body weight loss; TBWL, total body weight loss; EBMIL, excess body mass index loss
